# Prospective International Pilot Study Evaluating the Efficacy of a Self-Guided Contouring Teaching Module With Integrated Feedback for Transitioning From 2D to 3D Treatment Planning

**DOI:** 10.1200/JGO.18.00224

**Published:** 2019-05-13

**Authors:** Mustafa Abugideiri, Eduard Schreibmann, Jeffrey Switchenko, Mark W. McDonald, Jonathan J. Beitler, Walter J. Curran, Deborah Bruner, Pretesh Patel, Wondemagegnhu Tigeneh, Miressa Mijena, Sibo Tian, Anees Dhabaan, Natia Esiashvili, Tian Liu, Arif N. Ali

**Affiliations:** ^1^ Winship Cancer Institute of Emory University, Atlanta, GA; ^2^ Black Lion Hospital/Addis Ababa University, Addis Ababa, Ethiopia

## Abstract

**PURPOSE:**

Transitioning from two-dimensional to three-dimensional treatment planning requires developing contouring skills. Contouring atlases are excellent resources, but they do not provide users active feedback. Developing countries may not have many radiation oncologists experienced in three-dimensional planning to provide training. We sought to develop a standardized self-guided educational module with integrated feedback to teach contouring skills.

**METHODS AND MATERIALS:**

All 18 oncology residents at Black Lion Hospital/Addis Ababa University in Ethiopia were trained to contour the level II lymph node station. Residents took a baseline pretest quiz, survey, and contouring evaluation. Residents then watched an instructional contouring lecture and performed three additional cases with integrated feedback by comparing their contours to gold-standard contours. Residents then took a post-training quiz, survey, and contouring evaluation. Paired *t* tests and analysis of variance were used for analysis.

**RESULTS:**

Before training, the average number of total cases ever contoured was 2.4 and the average number of head and neck cases contoured was 0.5. Comfort with contouring improved from being “not at all comfortable” to “quite comfortable” after the 3-hour training (*P* < .001). The standard deviation between the resident contours and gold standard improved from 72.6 cm^3^ (pretest) to 7.4 cm^3^ (post-test). The average percentage overlap with the gold-standard contours and Dice similarity coefficient improved with each case performed, from 27.7% and 0.26 (pretest) to 80.1% and 0.77 (post-test), respectively (*P* < .001). After training, 16 of 18 (88.9%) residents produced a Dice similarity coefficient greater than 0.7, the threshold generally accepted for excellent agreement.

**CONCLUSION:**

This self-guided teaching module was an effective tool for developing level II lymph node contouring skills by providing active feedback and resulted in improved user confidence and accuracy compared with a gold standard. This module can be expanded to other disease sites and countries to further facilitate transitioning to three-dimensional treatment planning in developing countries.

## INTRODUCTION

With the advancement of technology in radiation oncology and more widespread installation of three-dimensional treatment planning equipment, there is increasing need for developing three-dimensional contouring skills. Despite the importance, standardization of volume delineation is still needed, and formal education on contouring is lacking and highly variable,^1-3^ especially in the developing world.

**CONTEXT**

**Key Objective** We developed and evaluated the feasibility and utility of a self-guided contouring teaching module with integrated feedback for the level II lymph node (L2LN) station in a developing country preparing to transition from two-dimensional to three-dimensional treatment planning.
**Knowledge Generated** We showed objective improvements in target delineation, anatomic knowledge, and user confidence. At completion, nearly all participants independently contoured the L2LN station with excellent agreement compared with gold-standard contours.
**Relevance** This protocol may offer an alternative for low-income countries to deploying their faculty members or residents internationally to learn contouring skills by providing a financially efficient and time-efficient mechanism for developing contouring skills.

Contouring may make up one of the largest sources of uncertainty in treatment planning.^1,4-8^ Inadequate target coverage can lead to inferior tumor control,^9^ and incorrect normal tissue delineation can lead to increased toxicity.^10^ Despite availability of consensus contouring atlases, significant contouring variability is still an issue, as demonstrated by more than 80% of all submitted contours requiring revision and more than 45% requiring multiple revisions for patients enrolled in RTOG 0529 (ClinicalTrials.gov identifier: NCT00423293).^11^

National and international cooperative groups and organizations have developed contouring atlases and seminars to help improve accuracy and standardize contouring.^1,2,12,13^ Contouring atlases are useful but do not provide their users active feedback. Some contouring training programs provide contouring feedback to users,^14,15^ and others do not.^3^ In addition, contouring feedback may be given at the end of the training module without additional examination to assess for improvement after feedback.^13^ It is a well-known concept in education that feedback is critical to more effective learning.^16,17^ Training sessions that included feedback have shown good success.^1,15^

Because of high clinic volumes and busy physician schedules, it is becoming more difficult for attending physicians to provide contouring feedback to residents, despite its critical role in the educational process. A recent study showed approximately one-third of surveyed residents rarely received feedback on their contours,^2^ and another showed less than 25% get formal contouring instruction.^3^

In developing countries with newly purchased modern equipment, there may not be many radiation oncologists experienced in three-dimensional planning to provide contouring feedback. Resources such as EduCase (RadOnc eLearning Center, Fremont, CA) offer excellent platforms where contours can be compared with a standard for feedback; however, these models are internet dependent.^2^ Because of insufficient internet bandwidth in nearly all developing counties, these Web-based programs would not be feasible. To help address the challenges transitioning from two-dimensional to three-dimensional treatment planning, this study seeks to evaluate a standardized, internet-independent, self-guided educational contouring module with real-time integrated feedback to facilitate this transition and help improve contouring skills in developing countries.

## METHODS AND MATERIALS

All 18 second-, third-, and fourth-year oncology residents at Black Lion Hospital/Addis Ababa University in Ethiopia were trained. The approximately 3-hour contouring training sessions were performed daily with two residents with the goal of learning how to contour the level II lymph node (L2LN) station. Head and neck (HN) lymph node (LN) stations were chosen because of their complexity, because HN contouring has been shown to have the greatest level of contouring variability^2,4,5^ and also because of the availability of a standardized contouring atlas.^12^ Residents performed a pretest survey that was modified from Gunther et al^13^ to assess baseline demographic data (Table 1). Residents were also given a pretest quiz on the anatomic boundaries of the L2LN and a post-test quiz to evaluate improvement (Table 1). Our primary objective was to improve contouring accuracy of the L2LN, and secondary objectives were to improve user confidence and knowledge of anatomic boundaries.

**TABLE 1:**
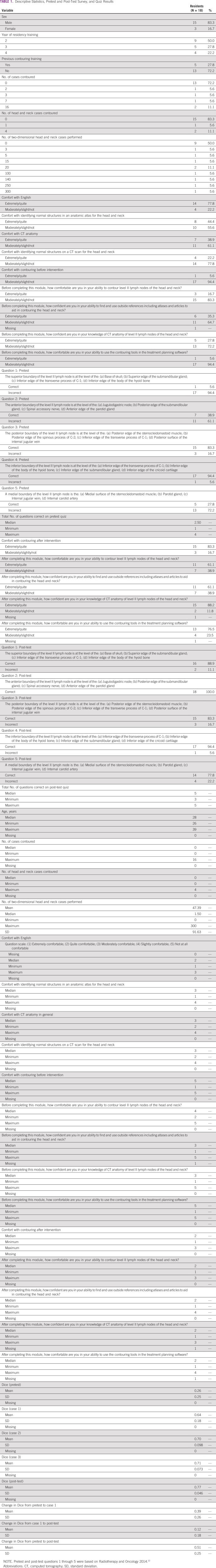
Descriptive Statistics, Pretest and Post-Test Survey, and Quiz Results

### Contouring Platform, Gold-Standard Contours, and Training Module Structure

Before training, a gold-standard L2LN contour was created. This contour was performed and verified by consensus of two US radiation oncology residents and two radiation oncology attending physicians against the international consensus guidelines.^12^ All residents contoured using VelocityAI (Varian Medical Systems, Palo Alto, CA). Residents gained familiarity with the software platform by contouring a vertebral body with instruction on how to use the contouring tools. Once residents stated they felt comfortable with the tools, they were instructed to contour every third slice of the right L2LN as a baseline. Residents had 20 minutes to complete the contours and were given 10-, 5-, and 2-minute warnings. Once completed, contours were interpolated. No additional edits could be performed after the time limit. No feedback was given after the first case.

Residents then watched an instructional L2LN contouring lecture from the EduCase Web site performed by Robert Amdur, MD.^18^ Residents were provided the HN LN consensus guidelines and atlas by Grégoire et al^12^ for reference during and after the video lecture.

After the lecture, residents contoured the right L2LN, as above, on the second case, with the guidelines and atlas as a reference. Once completed, they reviewed and compared their contours with the gold-standard contours on that specific case to get self-directed feedback, while also referring back to the atlas. Residents then performed two additional cases on the left L2LN, as above, with additional self-directed feedback on their contours after each case. After the fourth case, residents contoured the left L2LN without any resources as a post-test. A post-test quiz and survey were given to further assess the objective and subjective efficacy of the training. The pretest and post-test quiz questions were identical. Residents were given $25 gift cards on completion.

### Statistics

Resident contours were evaluated against gold-standard contours for accuracy using the Dice similarity coefficient (DSC).^19^ Average Hausdorff distance (HD),^20^ Jaccard,^21,22^ percentage overlap, false-negative error (FNE), and false-positive error (FPE) were also analyzed.^21,23^

DSC was estimated using the following formula:

$$DSC=\;\frac{2\;x\;\left(A\;\cap \;B\right)}{A+B},$$

where *A* is the volume of the expert/standard, *B* is the volume of the given rater, and *A* ∩ *B* is the intersecting overlap of the two volumes. DSC scores range from 0.0 to 1.0, with 0 representing no overlap and 1 representing perfect overlap. A DSC of 0.7 or greater represents excellent agreement.^24^ DSCs were measured at each time point, and change in DSC between pretest and post-test (Table 1), pretest and case 1 (Data Supplement), and case 1 and post-test were also estimated (Data Supplement). Paired *t* tests were used to assess for DSC differences and other similarity metrics between cases. Paired *t* tests were used to assess for differences in comfort level variables pre- versus postintervention, and McNemar tests were used to assess for change in correct quiz question response rates pre- versus postintervention.

**FIG 1:**
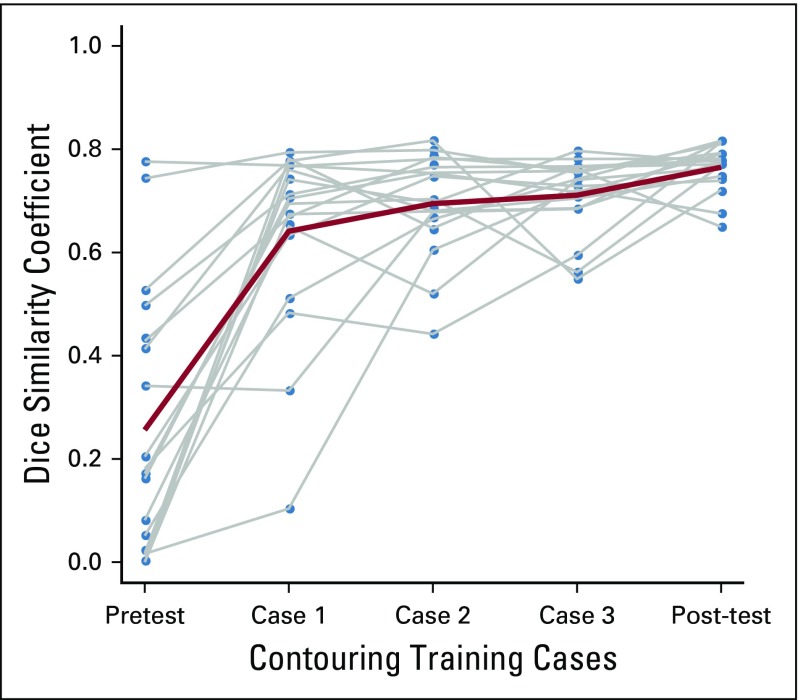
Plot of mean Dice similarity coefficient (DSC; red) and individual DSC of participants (gray) over time. Participants viewed a contouring didactic lecture between the pretest and case 1.

Resident characteristics, such as sex, age, year of residency, comfort with English, previous contouring training, number of cases contoured, number of HN cases contoured, self-reported comfort levels, and correct quiz question response rates, were summarized descriptively (Table 1). Resident characteristics were compared with change in DSC (post minus pre; Data Supplement) using analysis of variance or Kruskal-Wallis for categorical variables and Pearson’s correlation coefficient for continuous variables. In addition, resident physician characteristics were compared with pretest DSC (Data Supplement) and post-test DSC (Data Supplement). A plot of DSC scores across time was also produced (Fig 1).

Statistical analysis was performed using SAS 9.4 (SAS Institute, Cary, NC). The significance level was assessed at the 0.05 level.

## RESULTS

### Study Participants

Eighteen oncology residents from Black Lion Hospital/Addis Ababa University in Ethiopia completed the training module. The median age was 28 years (range, 26 to 39 years). Fifteen of the 18 participants (83.3%) were male. Nine (50%) were second-year residents, five (27.8%) were third-year residents, and four (22.2%) were in their fourth and final year of training. Five residents (27.8%) had previous contouring experience while performing an away rotation at another facility in Europe that used advanced three-dimensional treatment planning techniques; however, the average number of total cases ever contoured by all residents was 2.4, and the average number of HN cases contoured was 0.5. The median number of cases contoured was 0 for both total cases (range, 0 to 16) and HN cases (range, 0 to 4). Baseline demographic data are listed in Table 1.

### Contouring Volume Analysis

**FIG 2:**
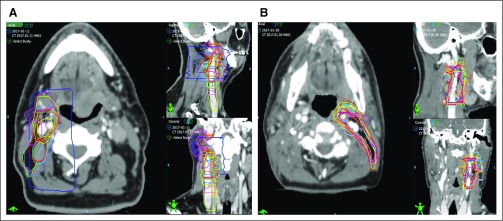
Selected slices of participant contours relative to gold standard (green). (A) Pretest contours. (B) Post-test contours.

**FIG 3:**
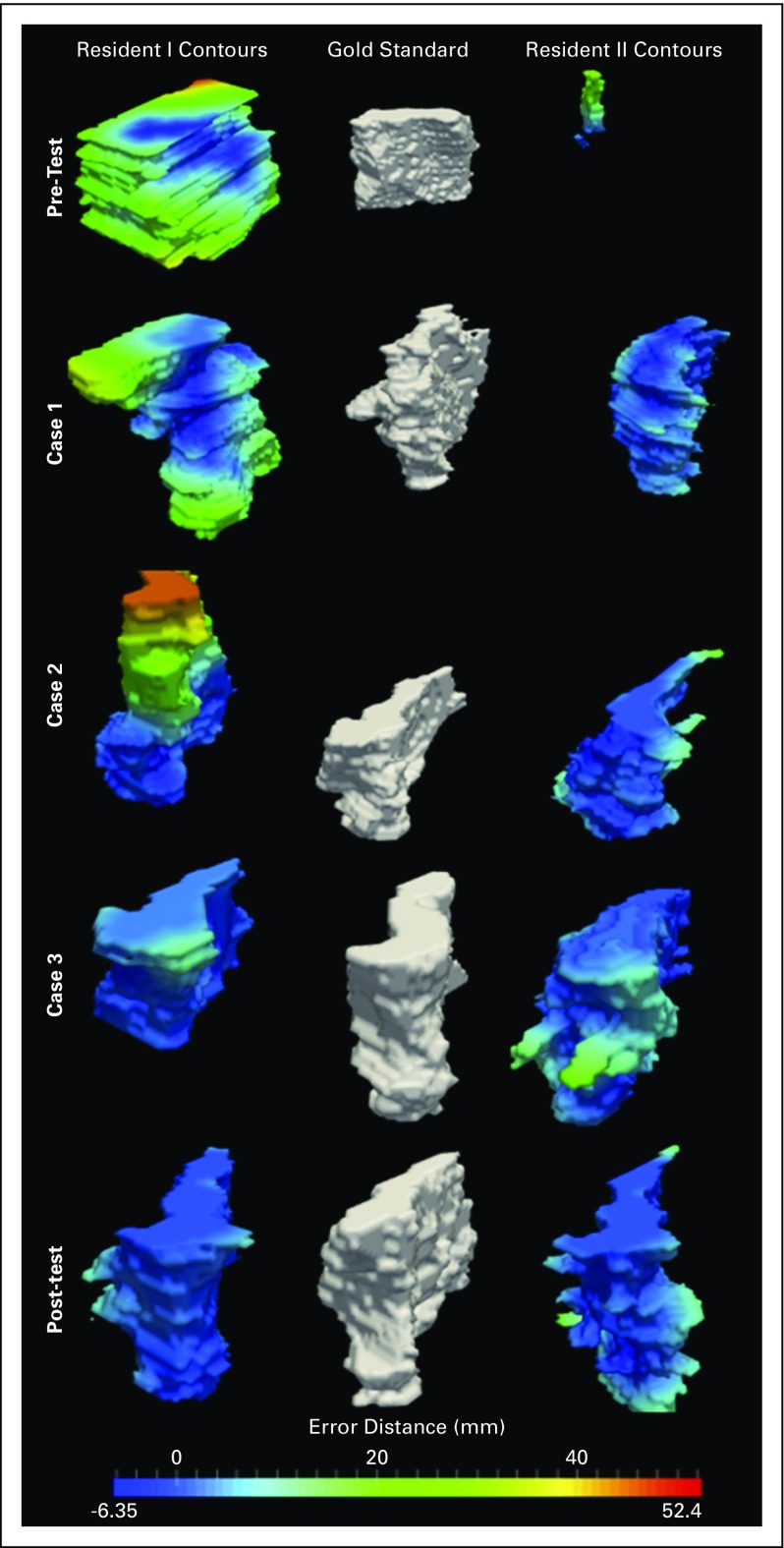
Demonstrates reduction in contouring error (millimeters) of resident sample cases (left and right images) relative to the gold standard (middle).

The standard deviation (SD) between resident contour volumes and the gold standard improved from 72.6 cm^3^ (pretest) to 7.4 cm^3^ (post-test). The percentage overlap with the gold standard contours and DSC improved with each case performed from an average percentage overlap of 27.7% and DSC of 0.26 on the pretest to 80.1% and 0.77, respectively, by the post-test (*P* < .001). After completing the module, 88.9% (16 of 18) of the residents produced a DSC greater than 0.7, and 87.5% of those residents (14 of 16) produced a DSC greater than 0.75. Other similarity measurement indices comparing pretest to post-test produced similar results, with mean HD improving from 43.7 to 17.4 mm, mean Jaccard improving from 0.17 to 0.62, mean FNE improving from 0.72 to 0.2, and mean FPE improving from 0.66 to 0.25 (*P* < .001; Table 2). Improvements in contouring are also illustrated in Figures 2 and 3.

**TABLE 2:**
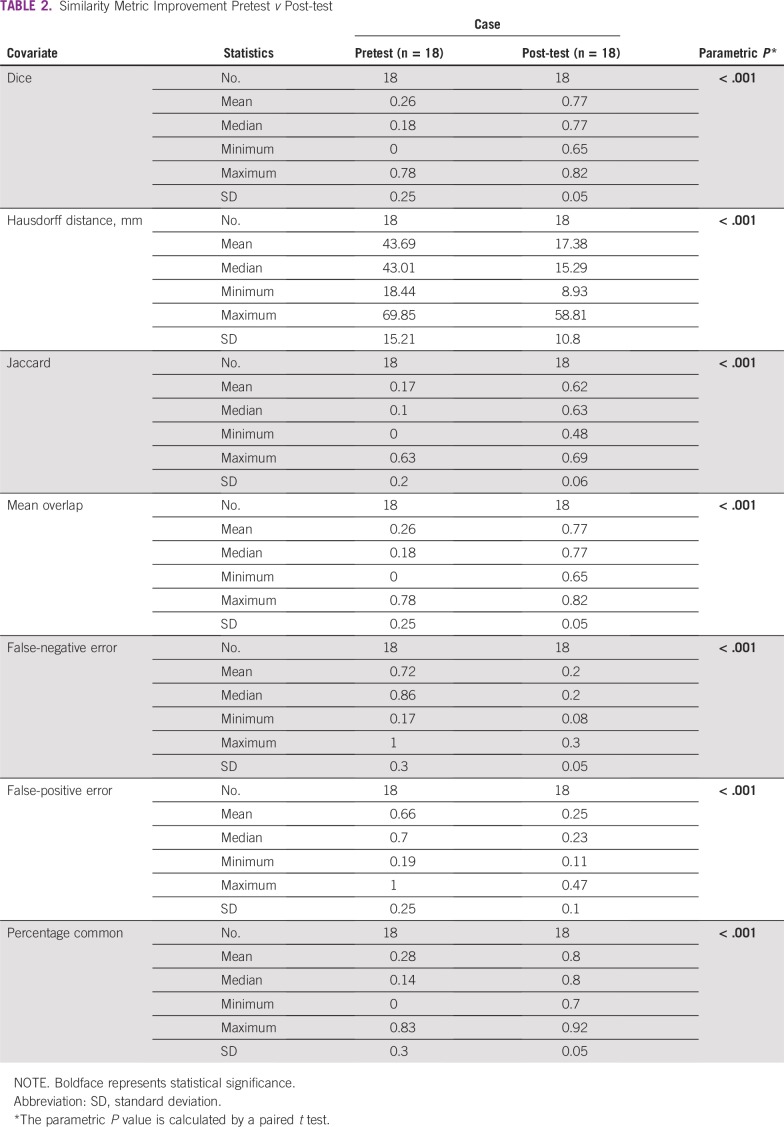
Similarity Metric Improvement Pretest *v* Post-test

Sex, year of residency training, comfort with English, self-reported comfort with computed tomography (CT) anatomy, comfort with LN anatomy, comfort with contouring in general, comfort with contouring L2LN, and previous HN contouring experience were not associated with differences in mean DSC improvement from pretest to post-test. There were, however, significant differences if residents had even limited previous contouring experience, with a mean DSC improvement of 0.59 for those without contouring experience compared with 0.29 for those with any contouring experience (*P* = .019; Data Supplement). On the contouring pretest, residents who did not have previous contouring training had a mean DSC of 0.17, which was lower than those who did have previous contouring training, with a mean DSC of 0.48 (*P* = .018; Data Supplement); however, this difference disappeared on the post-test, with a mean DSC of 0.77 for those with previous contouring training and 0.76 for those without it (*P* = .89; Data Supplement).

### Post-Training Evaluation

Resident confidence on the pretest with contouring in general and with contouring the L2LN improved from a median of 5 (not at all comfortable; range, 1 to 5) and 4 (slightly comfortable; range, 2 to 5), respectively, to a median of 2 (quite comfortable; range, 1 to 3) for both metrics after the teaching intervention (*P* < .001). On the pretest, only one resident self-reported feeling either extremely or quite comfortable with contouring, but this was likely erroneous, because they also reported no previous contouring experience. Residents were more comfortable using the contouring tools after the training, with an improvement in median scores from 5 (not at all comfortable; range, 1 to 5) to a median of 2 (quite comfortable; range, 1 to 4; *P* < .001). Statistically significant improvements were also noted in the self-assessed ability to find and use contouring atlases and comfort with CT anatomy of the L2LN stations after training (Table 3).

**TABLE 3:**
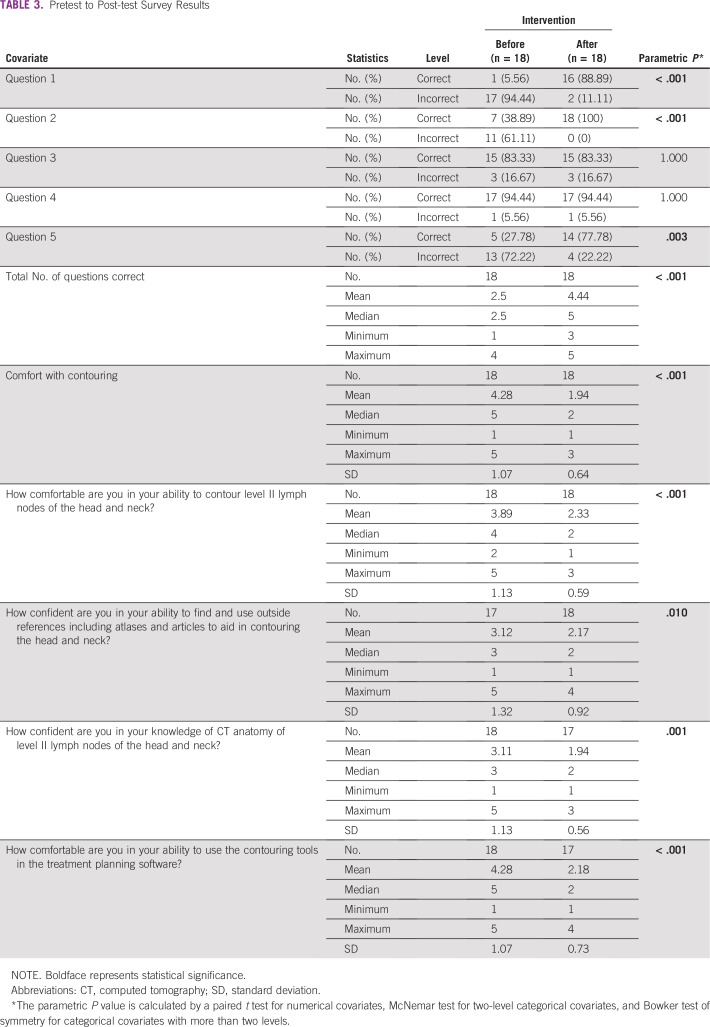
Pretest to Post-test Survey Results

Residents showed improvement in multiple choice quiz scores on the L2LN anatomic borders, with mean and median pretest scores of 2.5 questions answered correctly out of five questions, which improved to a mean of 4.4 and median of five questions answered correctly after the intervention (*P* < .001).

## DISCUSSION

We designed and evaluated a self-guided contouring module for the L2LN, which incorporated an instructional video lecture and three contouring cases with integrated feedback allowing users to compare their completed contours to a gold standard. After completion, improved user confidence in contouring and cumulative improvements in accuracy and decreased variability in contouring the L2LN were achieved. The greatest initial improvement occurred after the lecture, which provided a knowledge foundation to build on. The effectiveness of this protocol is underscored, given the fact that the majority of these residents had never contoured before and none had used the VelocityAI software.

Although long-term follow-up was limited to assess for lasting retention of skills and knowledge learned, the initial results of the study are quite impressive and promising. This protocol may offer an alternative for low-income countries to deploying their faculty members or residents internationally to learn contouring skills by providing a financially efficient and time-efficient mechanism for developing contouring skills. Completely automated software could be developed based on this teaching protocol. It could potentially be delivered to a country for on-site training, thus allowing all physicians in a department to be trained, instead of a select few. Because such automated software could be run any number of times on-site to achieve proficiency, physicians would not be forced to try to remember skills they learned when training in another country or go back for additional training. Feedback would be obtained on specific cases, as opposed to comparing the case at hand to a different case or contouring atlas and trying to extrapolate the results.

As part of a standardized curriculum, hospitals could acquire the software and training module. Once a certain level of proficiency was achieved, as determined by a contouring skills proficiency examination and objective examination of fund of knowledge, a certificate program could be developed.

With the limited feedback and lack of formalized contouring education domestically, this program could become a critical part of radiation oncology curricula. Gunther et al^14^ tried to address the lack of standardization in radiation oncology curricula by developing the introductory radiation oncology curriculum. They found that surveyed residents only felt slightly prepared for residency before their institutional orientation and moderately prepared on completing orientation. After completion of the introductory radiation oncology curriculum, more hands-on training with contouring was requested.^14^ A previous nationally delivered survey found only 11.3% of surveyed residents reported that their orientation was essential.^25^ This module could supplement orientation providing better preparation for residency and enhancing traditional apprenticeship-based teaching to help provide more consistent and standardized resident educational experiences.

HN LNs were chosen because of their intricate anatomy and increased level of contouring difficulty, with the notion that if residents were able to achieve success contouring such complex structures, then the success of the module would likely be reproducible with less difficult structures. A recent survey showed that up to 30% of surveyed US radiation oncology residents did not feel comfortable identifying normal HN anatomy, and approximately one-third did not feel comfortable identifying at-risk nodal sites.^2^ Another found only 27% of residents participating in an HN contouring seminar felt comfortable with target delineation before training.^26^ Sura et al^2^ showed that after undergoing an HN Webinar-based training, there was a nominal increase in user confidence in identifying the anatomy; however, it was not statistically significant.

In our study, residents showed objective, statistically significant improvements in multiple choice quiz scores on L2LN anatomic borders and contouring volumes assessed by DSC. Subjectively, user confidence in contouring the L2LN and CT anatomy of the L2LN also improved. This potentially supports the notion that incorporating feedback into the teaching protocol improves learning and, thus, confidence. Vega et al^27^ also showed that using an interactive contouring session resulted in nominal improvements in pretest to post-test quiz scores; however, contouring proficiency was not examined.

Awan et al^15^ used automated feedback as part of their training and showed statistically significant improvement in 16 of the 26 structures contoured. They noted a median DSC greater than 0.7 for four of 11 LNs after just one feedback session^15^ and may have produced even higher DSC if more feedback sessions were provided. We provided three opportunities for feedback; by the completion of the module, 88.9% (16 of 18) of the residents produced a DSC greater than 0.7, and 87.5% of those residents (14 of 16) produced a DSC greater than 0.75. A DSC greater than 0.7 corresponds with excellent agreement on the basis of the literature.^24^ Although De Bari et al^1^ showed improvement using feedback incorporated into their teaching model, it is difficult to discern the level of success, considering the preintervention DSC for their gross tumor volumes and clinical target volumes were both greater than 0.7. In addition, all contours were performed on the same case, which may have affected their results, whereas our study used five distinct cases. Another study showed improvement in contouring skills without dedicated feedback; however, only two of 18 of the post-test structures produced an average DSC greater than 0.7, and only one was statistically significant.^3^

Differences in contouring may make up one of the largest sources of uncertainty in treatment planning,^1,4,5^ although training can reduce variability.^1,6,26,28-30^ Because of this, improvements in FPE and FNE are even more significant. FPE represents areas that are labeled positive by the user, but not the expert, whereas FNE measures areas deemed positive by the expert but missed by the user.^23^ FPE would likely result in increased toxicity, because an area not deemed to be at risk by the expert was targeted for therapy; FNE would likely result in decreased tumor control, because a critical area that required targeting for therapy was missed. Both of these metrics showed statistically significant improvement from pretest to post-test, with a larger improvement in FNE, arguably the more important of the two.

DSC and Jaccard are the most commonly used measures of spatial overlap.^23^ HD is commonly used but is more susceptible to outliers.^23^ Because there is no consensus on the optimal measure or a standard method to evaluate interrater reliability,^31^ we used a combination of statistical measures of agreement and overlap to provide to most clear picture of the accuracy and variability in target volume delineation. This makes our study unique, with the usage of numerous similarity metrics. Because contours were compared with a gold standard, our main focus was the DSC.

Limitations of this study include selection bias to those at Black Lion Hospital/Addis Ababa University and small sample size. Despite survey scores indicating residents were quite comfortable with English, there were likely some imperfections in question interpretation due to language. In addition, there was not a control group to see whether different teaching methods would produce similar results. Although a statistically significant improvement in DSC was also noted between case 1, when feedback was initiated, and the post-test (Table 1; Data Supplement), it was not as drastic as the improvement between the pretest and case 1, when residents watched the instructional lecture, making it difficult to discern whether the feedback or the lecture was more critical to learning or whether the entire module was essential. Our main objective was to evaluate the teaching module holistically, with both the didactic portion and feedback; however, future study could examine whether implementing feedback before the lecture, or without a lecture altogether, would produce similar results. We were also limited in our ability for long-term follow-up with testing for retention of knowledge and skills learned and assessment of the impact on clinical practice.

This study demonstrated the successful implementation of a self-guided contouring teaching protocol with standardized didactic lectures and integrated contouring feedback. Using this teaching intervention in a developing country is feasible and associated with objective improvements in L2LN target delineation, anatomic knowledge, and user confidence. After completion, nearly every participant was able to independently contour the L2LN station with excellent agreement with the gold standard. Future work will be to develop fully automated, internet-independent software on the basis of this protocol and include gross tumor volumes with validation across all disease sites and expansion to other training locations with long-term follow-up. We will continue to explore using this protocol as a teaching tool internationally and domestically.
